# Waterborne Random Copolymers as Polymeric Surfactants for Emulsion Polymerization

**DOI:** 10.1021/acs.iecr.5c04814

**Published:** 2026-01-30

**Authors:** Aitor Barquero, Miren Aguirre, Jurgen Scheerder

**Affiliations:** † POLYMAT and Kimika Aplikatua Saila, Kimika Fakultatea, University of the Basque Country UPV/EHU, Joxe Mari Korta Zentroa, Tolosa Hiribidea 72, 20018 Donostia-San Sebastián, Spain; ‡ 644881 Covestro (Netherlands) B.V., Sluisweg 12, 5145 PE Waalwijk, The Netherlands

## Abstract

Waterborne acrylic polymer dispersions are typically produced via emulsion polymerization by using low molar mass surfactants. While these surfactants ensure dispersion stability, they negatively affect coating properties, such as water resistance, adhesion, haze formation, and barrier performance. A promising alternative is polymeric surfactants. Properly designed, polymeric surfactants prevent leaching and associated issues while enhancing coating performance. This review begins by explaining the use of polymeric surfactants, followed by an overview of their types and the polymerization methods employed—primarily bulk, solution, and emulsion polymerization. Then, polymeric surfactants synthesized through emulsion polymerization will be described, and their role in subsequent emulsion processes for waterborne binder production will be discussed. Additionally, the review examines coating morphology and presents examples of industrial applications, including coatings for printing and packaging.

## Graphical abstract

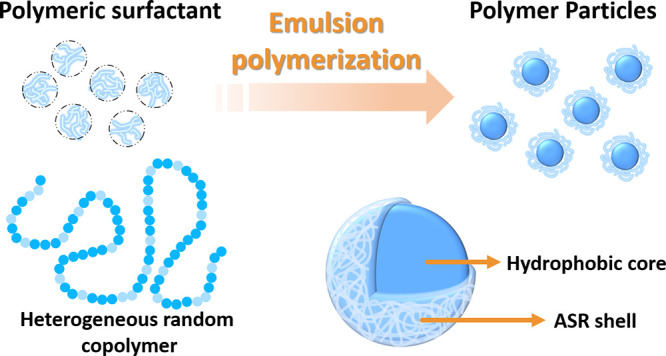

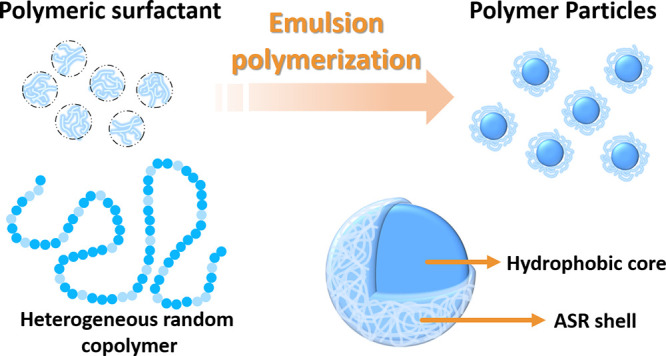

## 1 Introduction

Waterborne polymer dispersions prepared by emulsion polymerization are widely used in the coatings and inks industry. Emulsion polymerization is a very versatile process that allows developing waterborne acrylic binders with unique properties.^1^ For instance, the synthesis of dispersions with different polymer phases allows the design of waterborne acrylics with a multitude of particle morphologies.^2–4^ The inherent versatility of the binder system allows the combination of different properties, which is achieved through the incorporation of different polymer phases. This enables the simultaneous achievement of antagonistic properties such as high hardness and low coalescence demand, for instance. Another advantage of using emulsion polymerization is that water is used as the continuous phase, making the process environmentally friendly not only because the need for volatile organic compounds (VOC) is drastically reduced but also because the energy generated by the exothermic radical reactions can be used to heat the reactor content.^5^

One of the essential ingredients of waterborne acrylic polymers is low molar mass surfactants, which are critical for the preparation and stabilization of the colloidal dispersion. Surfactants are crucial during the nucleation and growth of the polymer particles, as well as to provide sufficient colloidal stability to ensure proper storage and transport stability.^6^ However, during the film-forming process of waterborne dispersions, the low molar mass surfactants are associated with leaching to the interphase between the polymeric film and air, reducing the final properties of the polymeric film, such as water resistance,^7^ water whitening,^8^ reduced moisture vapor transmission,^9^ and loss of adhesion.^10^

To reduce or avoid the negative effects of low molar mass surfactants, in the last decades, various approaches have been developed, such as the synthesis of surfactant-free emulsion polymers^11^ and the use of copolymerizable surfactants^12^ and polymeric surfactants.^13^ In the literature, several works in which surfactant-free emulsion polymerizations were used have been reported, but there are some limitations. The selected comonomers have to be ionic or charged to provide sufficient colloidal stability, achieving low solids contents in most of the cases^14,15^ and difficulties to synthesize small particle sizes in the range of 50–300 nm.^16^ Nonetheless, there are some examples of high solids contents for anionic^17^ and cationic^18^ systems. On the other hand, the use of copolymerizable surfactants has also been studied quite extensively.^19,20^ These low molar mass molecules are surface active and act as surfactants; however, they will also be chemically bound to the polymer particles and cannot migrate through the polymer film to the surface freely, improving properties such as water resistance and adhesion.^21,22^ Nevertheless, there are several issues associated with the use of copolymerizable surfactants. The reactive groups of these surfactants are often different from the ones of common monomers, which may lead to vastly different reactivity ratios and poor incorporation or even homopolymerization of the copolymerizable surfactants in the water phase, which is not desired. Alternatively, if the incorporation is good, the resulting dispersion might have high surface tension due to the lack of free surfactant, which impairs wetting, leveling, and the formulation latitude of the dispersion.

The use of polymeric surfactants, often also termed alkali-soluble resins (ASRs), is another approach to mitigate the disadvantages of low molar mass surfactants. Polymeric surfactants find widespread use in oil recovery, pharmaceutical applications (antimicrobial, drug, and gene delivery), cosmetic and personal care (as stabilizers and thickeners), paper making (improving paper performance and ink removal), water treatment, and the textile industry (wetting, defoaming...).^20,23,24^

There are multiple benefits of using polymeric surfactants over traditional low molar mass ones. Due to their higher molar mass, there will be little to no tendency to exude from the polymeric film when the binders are used for coatings, for instance. Figure 1 shows the undesired haze created by surfactant exudation or leaching from an acrylic coating when the traditional, low molar mass sodium dodecyl sulfate (SDS) was used as the surfactant.^25^ Moreover, the higher molar mass of the polymeric surfactants offers diverse advantages for application areas where the presence of low molar mass molecule’s fractions (with $\overset{®}{M_{w}\;}$ <1000 and <500 g/mol) causes concerns regarding regulatory issues, such as printing and food packaging, cosmetic, personal care, and medical applications.

**Figure 1:**
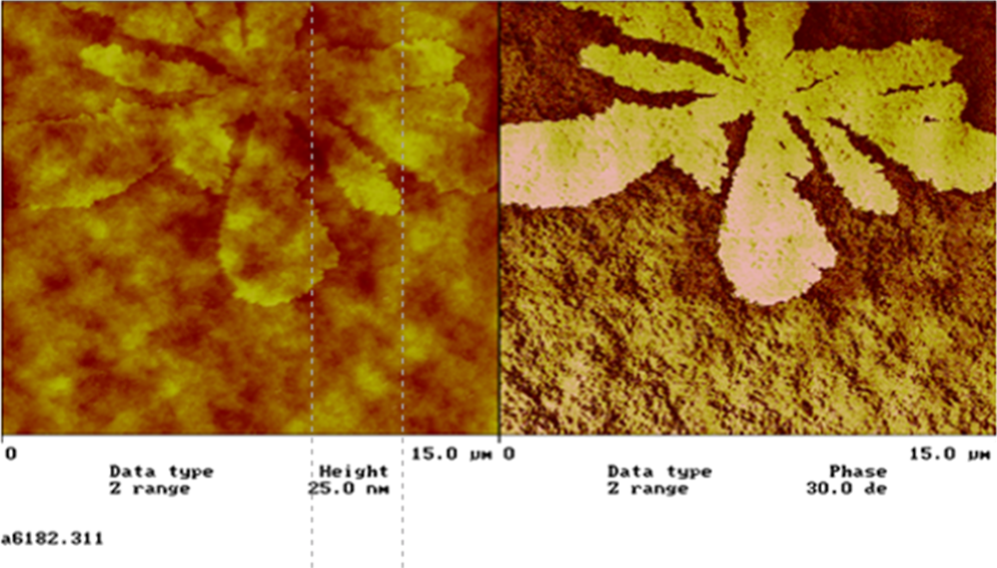
An example of surfactant exudation from an acrylic coating. Left, height image, and right, phase image. The lighter feature represents the exuded surfactant. Reproduced with permission from ref 25. Copyright 2001 Vincentz Network and www.european-coatings.com.

The greatest benefit of polymeric surfactants is the large degree of freedom to design them. By playing with their composition, characteristics such as the glass transition temperature (*T*_g_), functionality (acid value, cross-linking groups...) and hydrophilic–lipophilic balance can be easily tuned.^24^ On the other hand, molecular parameters such as molar mass and structure (linear, branched, random, block, etc.) can also be manipulated, contributing to enhancing the final properties of the polymeric films.

These polymeric surfactants have been a key advancement that has led to the development of a whole range of binders for the coatings and ink industry with significant commercial impact. The technology of waterborne binders, based on polymeric surfactants made via emulsion polymerization, has been pioneered by many companies and supported by the extensive patent literature. Some of these companies include Mitsubishi,^26^ S.C. Johnson & Son,^27^ the Rohm and Haas Company,^28^ BASF,^29,30^ and Covestro.^31,32^ Alongside this technology, polymeric surfactants made via solution or bulk polymerization have also been developed by companies including S.C. Johnson & Son,^33^ American Cyanamid,^34^ Henkel,^35^ Rohm and Haas Company,^36^ and BASF^37^ among others.^38^ It should be mentioned that polymeric surfactant-based binders prepared via mini-emulsion polymerization have also been described in the open literature.^39^

When polymeric surfactants are used as an alternative to low molar mass surfactants, their colloidal properties are critical. Their surface activity and ability to stabilize an oil or polymer phase is a prerequisite to being an alternative to low molar mass surfactants. In addition to this, their structure and composition can also positively impact the final properties of the binders, coatings, and inks derived from them. In industrial coating applications on wood (furniture and joinery), these binders enable achieving a balance between coating properties that are seemingly opposing each other. For example, it allows designing hard coatings that provide scratch resistance and antiblocking behavior, combined with a reduced coalescent demand, good water resistance, and high flexibility. The polymeric surfactant plays an essential role in achieving this balance since they are used in much higher amounts than low molar mass surfactants. Thus, their *T*_g_, molar mass, and composition contribute to coating properties such as hardness, antiblocking, scratch resistance, and water resistance. In addition, in cases where the polymeric surfactants are hydrophilic in nature, they contribute during film formation via water plasticization, reducing the need for coalescing agents and allowing defect-free film formation. In printing inks, polymeric surfactants provide much better reversibility and wet and dry adhesion compared to those of low molar mass surfactants. In all of these applications, these positive effects cannot be achieved with low molar mass surfactants.

In this review, the different approaches that have been used in the last years regarding the synthesis of polymeric surfactants will be summarized. In addition, the main goal of this review is to gather knowledge on the synthesis of the polymeric surfactants by emulsion polymerization and its further incorporation in a second step by emulsion polymerization as well. It has been demonstrated by several authors that this path offers not only flexibility to synthesize the polymeric surfactants but also improvements in the final application of the polymeric film. Mainly coating application will be discussed.

## 2 Synthesis of Polymeric Surfactants

As previously mentioned, in order to replace low molar mass surfactants during the synthesis of waterborne polymeric dispersions, the polymeric surfactants must be surface active. The polymer structure is particularly important for polymeric surfactants because the surface activity is the consequence of the heterogeneity of the chains since they must be able to organize themselves at an oil–water interface. Acrylic polymeric surfactants can be prepared via radical or controlled radical polymerization (CRP) techniques. Typically, CRP is carried out in homogeneous media (solution or bulk polymerization), while radical polymerization can be carried out by either homogeneous or heterogeneous polymerization (emulsion). Therefore, the surface activity not only depends on the composition (monomer selection, molecular weight, *T*_g_, acid value...), but also on the polymerization method used to prepare them. Further details are available in a recent review from Aleid and co-workers.^24^ Figure 2 gives an overview of the various polymerization methods and their impact on the polymer backbone structure and the surface activity of the polymeric surfactants.

**Figure 2:**
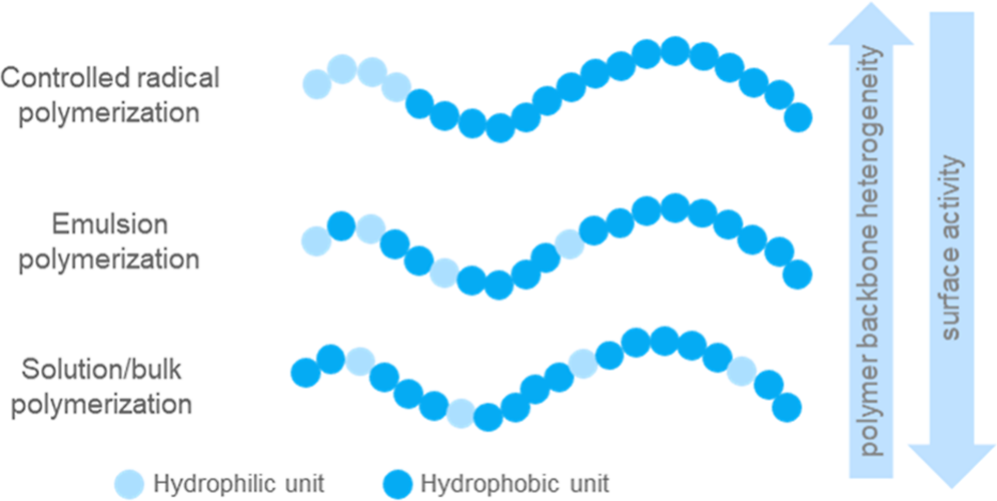
Overview of various radical polymerization methods used to make acrylic polymeric surfactants and their impact on polymer backbone structure and surface activity.

In the following section, the effect of the polymerization method on the molecular structure and emulsifying capacity of the polymeric surfactants will be discussed.

### 2.1 Polymeric Surfactants Made by CRP

CRP techniques offer a very high level of control over the polymer backbone structure and have been used to prepare (multi)­block copolymers with well-controlled block compositions and block lengths in dispersed systems.^40–42^ Due to their heterogeneous character, such (multi)­block copolymers are excellent polymeric surfactants to be used in either (mini-)­emulsion polymerization or suspension polymerization. The self-assembly of the preformed block copolymers, which is a first step in their use as polymeric surfactants, was described by Lutz.^43^ It was also demonstrated that CRP techniques could produce structures that self-assemble in aqueous media. This so-called polymerization-induced self-assembly process (PISA), depending on the structure of the block copolymer, can produce a whole range of particle morphologies^44^ and has already been used for the polymerization of common monomers such as styrene (S) and methyl methacrylate (MMA).^45,46^ PISA is a very extensive topic that has already been deeply reviewed elsewere^47,48^ and the topic will not be further discussed in this review.

However, two aspects hinder the widespread use of CRP techniques on an industrial scale. The first aspect is that many CRP techniques require advanced synthetic methods that are often not compatible with the existing industrial polymerization processes. This significantly limits their applicability. The second aspect relates to the high level of control that CRP techniques offer, especially precise control over the location of functional groups. Functional groups such as acid, hydroxyl, ketone, acetoacetoxy, tertiary amine, or cationic charges are used to introduce specific properties such as adhesion, cross-linking, chemical resistances, or durability, which contribute significantly to the final coating performance. When CRP is used to prepare block copolymers, often the functional groups are located in a single block. However, this may not be the most effective way to incorporate functional groups. For instance, when all cross-linking groups are located in one block, most of these will not be involved in the further cross-linking reaction, as the first cross-link reaction will increase the glass transition temperature, reducing the mobility of the chains and restricting the accessibility of the neighboring functional groups.

Interestingly, for many industrial applications, a high level of control typically achieved by CRP techniques is not necessary. One recent development is to use block–random copolymers (BRCs) as polymeric surfactants, which was pioneered by Cunningham and co-workers.^49^ In their approach, a first block is prepared using nitroxide-mediated polymerization, followed by extension with S and acrylic acid (AA). These BRCs were shown to self-assemble into aggregates consisting of multiple chains^50–52^ that were effective polymeric surfactants in emulsion polymerization.^53^ This technique can be considered to bridge the gap between polymeric surfactants made by CRP techniques and other radical polymerization methods.

All of these polymerization techniques have one element in common; they all produce polymers with a heterogeneous structure that exhibit high surface activity and are very suitable to be used as polymeric surfactants. However, the heterogeneity does not have to be perfect, and this is where emulsion polymerization comes into play.

### 2.2 Polymeric Surfactants Produced by Radical Polymerization

Radical polymerization techniques offer multiple tools to manipulate the structures of polymers. The main advantage over most CRP techniques is that the former are routinely applied on an industrial scale. It allows designing the chemical composition and the introduction of functional groups that promote adhesion and provide colloidal stability, cross-linking points, as well as allowing control of the molar mass, ranging from a few thousand to millions. Furthermore, when the reactions are carried out in heterogeneous media, it is possible to tune the particle morphology and particle size (and distribution) of the final polymer chains. By altering these parameters, a wide variety of polymeric surfactants in terms of composition, functionality, molar mass, and *T*_g_ can be made. This also opens possibilities to provide further properties beyond their surface activity. One example is the introduction of cross-linking into polymeric surfactants. In particular, the introduction of self-cross-linking technology which resulted in polymeric surfactants that had a significant contribution to improving the final mechanical properties of the coating.^25,54^

Emulsion polymerization is a heterogeneous process and results in polymeric surfactants with a rather broad distribution in functionality, molar mass, particle size, and polarity. In particular, the incorporation of acid functional monomers tends to be heterogeneous, leading to a distribution of acid groups and the formation of water-soluble material.^55–59^ Although an individual polymer chain produced via emulsion polymerization may exhibit limited surface activity, the composition of the overall formulation can be highly surface active and could be effectively used as surfactants. The micelle-like structures formed by these polymers will be heterogeneous in nature, comprising a diverse array of polymer chains that vary in functionality (for instance, acid value), molar mass, and polarity. The importance of polymer heterogeneity in waterborne coatings is well documented.^60^

In contrast, polymeric surfactants made via solution (or bulk) polymerization tend to be rather homogeneous in functionality, molar mass, and polarity. As a result, these tend to be less surface active, making it more complicated to use them as polymeric surfactants. Therefore, when they are incorporated as a surfactant in an emulsion polymerization step, often a semibatch process is used where the monomer and the polymeric surfactant are fed during time. In literature, most of the works used alkali-soluble resins composed of S, α-methylstyrene (α-MS), and AA, optionally with an acrylate ester to tune the *T*_g_. These are commercially available with weight-average molar masses between 2500 and 15,000 g/mol, acid values between 100 and 260 mg KOH/g, and *T*_g_ between 70 and 130 °C.

The use of solution-borne polymeric surfactants in emulsion polymerization was extensively studied by Kim and Lee.^61^ They studied the emulsion polymerization of styrene in the presence of an S/α-MS/AA polymeric surfactant with an acid value of 190 mg of KOH/g using calorimetry to monitor the heat of reaction. They extended their studies toward other monomers, including *n*-butyl methacrylate (BMA), ethyl methacrylate, and MMA.^62–65^ They observed a decrease in the rate of polymerization when a polymeric surfactant was compared with a traditional low molar mass surfactant. In addition, they observed a decrease in the rate of polymerization when the concentration of the polymeric surfactant was increased and also when the degree of neutralization (DN) was higher. This was attributed to the presence of a thicker and denser layer of the surfactant on the particle’s surface, and as expected, when more polymeric surfactant was used, a smaller polymer particle size was obtained. In addition to the S/α-MS/AA polymeric surfactant, styrene–maleic anhydride copolymers (SMA) have also been used. Modification with long-chain alcohols made them more surface active, resulting in 38–59 nm polymer dispersions.^66^ Nzudie et al., measured the critical micellar concentration (cmc) of polymeric surfactants with different styrene-maleic anhydride ratios, and they observed that increasing the styrene ratio, and hence the hydrophobicity, the cmc was decreased.^67^ The polymeric surfactant with an equal ratio between the S and MA was the only one able to emulsify the system and obtain stable latexes. However, very recently, Deng and co-workers have been able to synthesize anisotropic peanut-shaped PS particles through emulsion polymerization using SMA as a copolymeric surfactant, adding a small amount of sodium dodecylbenzenesulfonate.^68^

Cationic polymeric surfactants have also been synthesized, and the difference in surface activity between two identical polymeric surfactants in overall composition, one made via emulsion polymerization and the other via solution polymerization, was studied.^69^ This study showed that the polymeric surfactant made via emulsion polymerization presented higher surface activity. In aqueous medium, aggregates from the emulsion polymer were more condensed, while the aggregates from the solution polymer responded more strongly to the salt addition.

Other aspects, such as the grafting of polymeric surfactants, have also been studied by several authors, in particular because of the presence of styrene in these polymeric surfactants that makes hydrogen abstraction likely to occur and the presence of a terminal double bond formed during their synthesis.^70–73^ Increased grafting was observed when more polymeric surfactant was used and at high initiator concentrations. Interestingly, Bandiera et al. found that the incorporation via the terminal double bond was the dominant mode of producing grafting during the emulsion polymerization process.^73^

Polymeric surfactants were also used to prepare high-solid latexes via emulsion and mini-emulsion polymerization. By reducing the polymeric surfactant amount below the commonly used ones, a solid content of 57% was reached.^71^

## 3 Use of Polymeric Surfactants

Up to this point, the different synthesis methods of polymeric surfactants by emulsion polymerizations have been discussed, but their utilization in a subsequent emulsion polymerization process has also been extensively documented in the literature.^74,75^

The process by which polymeric surfactants are made and used in emulsion polymerization is shown in Figure 3.

**Figure 3:**
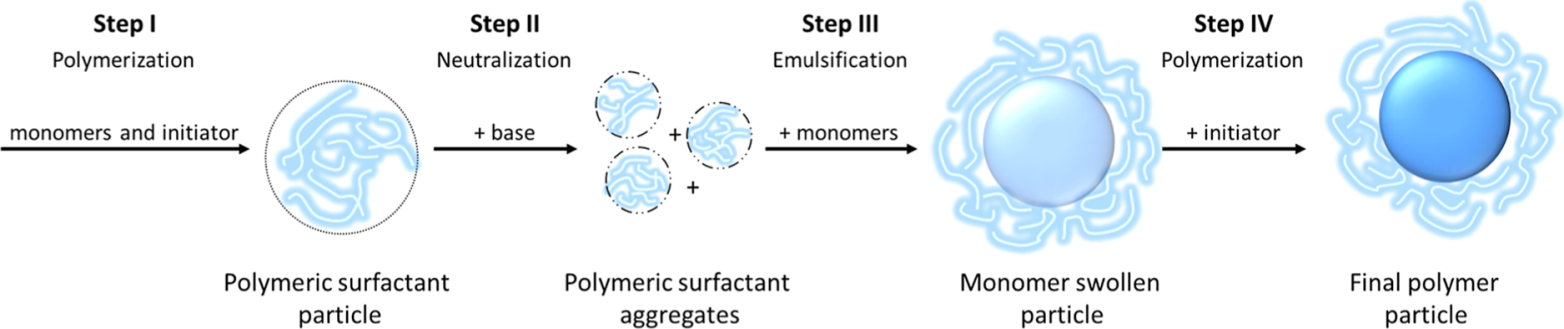
Process of preparing and the use of polymeric surfactants in an emulsion polymerization process. The polymeric surfactant chains are indicated in light blue, the polymer phase prepared in its presence is indicated in dark blue.

For polymeric surfactants produced by emulsion polymerization, this process comprises four main steps: (I) synthesis of the polymeric surfactant, (II) neutralization, (III) emulsification of monomers, and (IV) polymerization. In step (I), emulsion polymerization is used to synthesize the polymeric surfactant, which typically has a relatively high acid value (wt % acid between 5 and 50 wt %) and a low molar mass ( $\overset{®}{M_{w}\;}$ between 5000 and 50,000 g/mol). In step (II), a base is added, and the polymeric surfactant partially dissolves and becomes surface active. In step (III), monomers are added and emulsified by the polymeric surfactant before step (IV), where the monomers are polymerized in the presence of the polymeric surfactant. This step can be done following a batch or semibatch process.

The process by which solution polymeric surfactants are used differs from that shown in Figure 3 mainly because the polymeric surfactants made via solution or bulk polymerization are synthesized at much higher temperatures and pressures than in emulsion polymerization. This is so because once the resulting polymers are synthesized, they are neutralized and dispersed into water to proceed with the emulsion polymerization process. It should be mentioned that in most cases, the solvent is also removed.^33–38^

One of the main differences when comparing a typical formulation of emulsion polymerization is that the amount of polymeric surfactant used is much larger compared to that of traditional surfactants. The latter are typically used in amounts between 0.5 and 3 wt % (on solids), while polymeric surfactants typically are used in amounts of 10–50 wt %. This means that the polymeric surfactants are substantial parts of the final coating.

### 3.1 Neutralization and Solubilization

The effect of the acid value, DN, type of base, and molar mass of the polymeric surfactants synthesized via emulsion polymerization on the neutralization step was investigated by multiple authors.^76–80^ This step is essential, as it renders the polymer surface active. Figure 4 shows the effect of the DN on the solubility of the two MMA/BA/MAA random copolymers made via emulsion polymerization. The weight fraction of methacrylic acid (MAA) was 4 or 16 wt %, and the molar mass was 10,000 or 40,000 g/mol, which are representative of polymeric surfactants.

**Figure 4:**
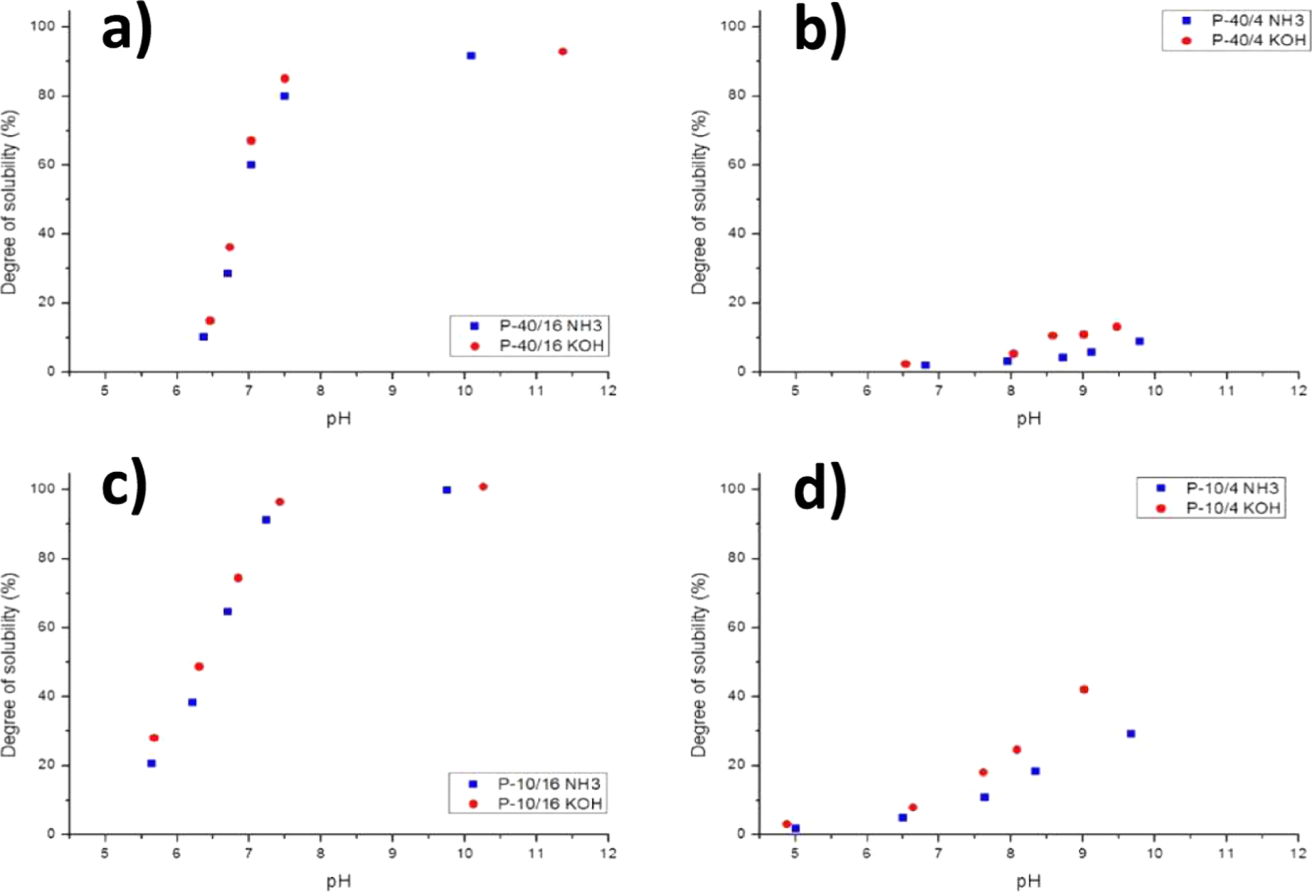
DS as a function of the pH and type of base. (a) Curve for P-40/16, (b) P-40/4, (c) P-10/16, and (d) P-10/4. The blue squares indicate the DS with ammonia, and the red dots indicate the DS for KOH. Reproduced with permission from ref 74. Copyright 2018 John Wiley and Sons.

As shown in Figure 4, the amount of acid is more important for the solubility than the molar mass. Both polymers with 16 wt % MAA show a high (>90%) degree of solubility (DS) at high pH (>7.5), whereas both polymers with 4 wt % MAA show solubility well below 50%. Using KOH results in a higher DS compared to using ammonia, which can be explained by the expected higher solubility of the potassium carboxylate salt in comparison with the ammonium salt. When the acid value is high and the molar mass is low, the DS almost reaches 100% (P-10/16), while when the acid value is low and the molar mass is high, the DS does not exceed 14% (P-40/4). These results are in line with earlier work from Muroi et al.^76^ In their work, the solubility in alkaline media was shown to depend on the wt % of acid groups present, the *T*_g_, the hydrophilicity of the polymer backbone, the molar mass, the chain configuration, and the temperature of dissolution. In additional work, they used potentiometric and conductive titration to study random copolymers of ethyl acrylate (EA) and MAA or AA, and their work showed that upon increasing the pH, the surface layers of the particles began to dissolve.^77^

When adding a base to acid-containing copolymer dispersions, the particle size increases due to deprotonation of the carboxylic acid groups. Due to the formation of COO^–^–NH_4_^+^ or COO^–^–K^+^ moieties, particle swelling with water increases. This effect is expected to become stronger as more base is added, as can be seen in Figure 5.

**Figure 5:**
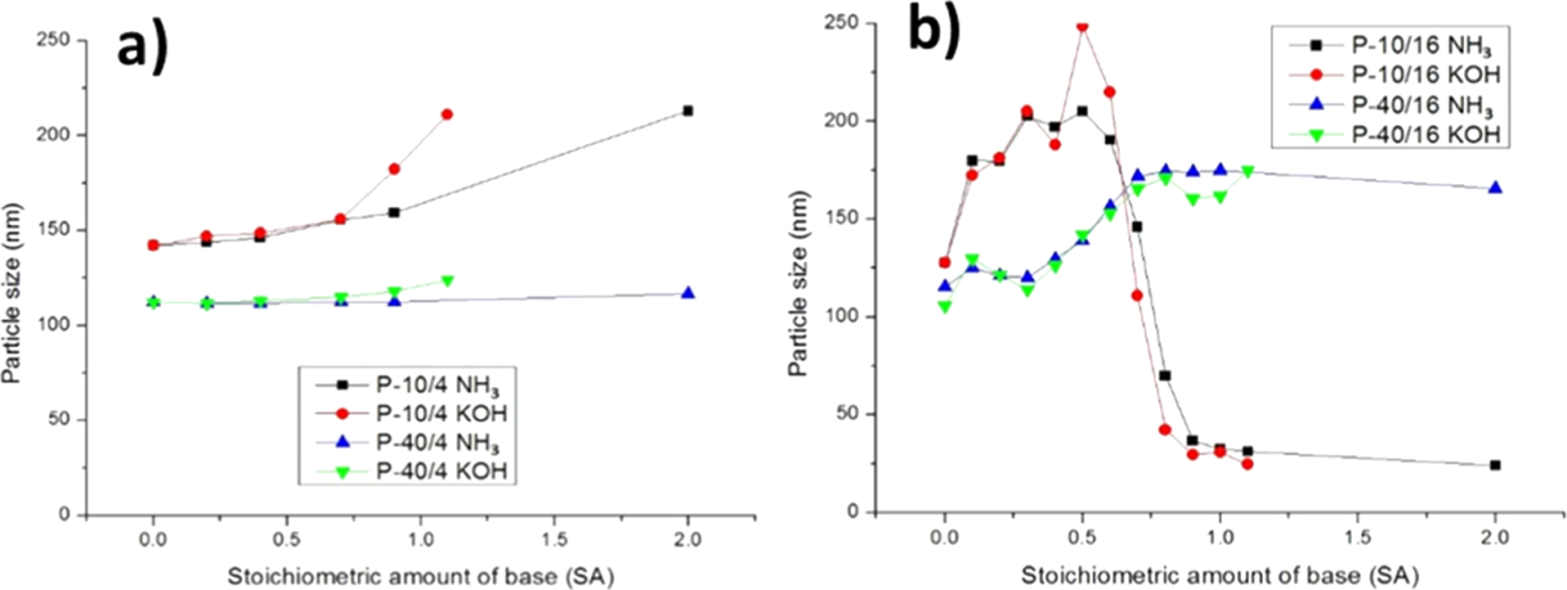
Particle size as a function of the amount of ammonia and KOH measured with DLS. Left: the two model systems containing 4% MAA. Right: the two model systems containing 16% MAA. Reproduced with permission from ref 74. Copyright 2018 John Wiley and Sons.

It can be seen that upon increasing the amount of base, the particle size increases, which indicates particle swelling. The extent of swelling depends on the amount of acid and the molar mass of the polymeric surfactants. When the amount of acid is high enough, the swelling of the particles is followed by disaggregation of the polymer chains due to their increased solubility. This mechanism of swelling and dissolution is in line with the work by Bassett et al.,^79^ Verbrugge,^78^ and Siddiq et al.^80^ On the one hand, Basset and coauthors studied hydrophobic alkali-swellable emulsions and followed the behavior of the polymer upon adding base using dynamic light scattering (DLS) and fluorescence. On the other hand, Verbrugge concluded that the amount of MAA and polymer *T*_g_ and hydrophilicity were the most important parameters. In addition, Verbrugge also studied this process using light microscopy and was able to visualize the swelling and dissolution. Interestingly, they reported that no difference was observed between using NaOH or ammonia as the base. More recently, this process was also studied by Cryo-TEM, as shown in Figure 6.

**Figure 6:**
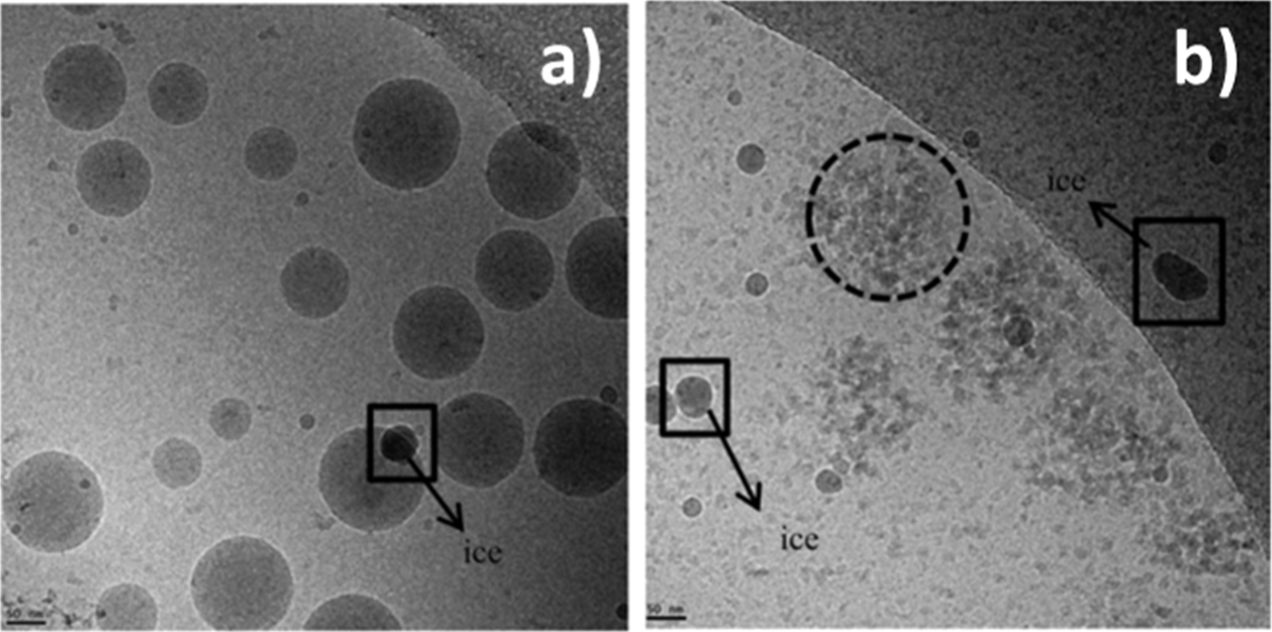
Cryo-TEM images of P-10/16 at (a) 0 SA ammonia and (b) at 0.7 SA ammonia (right). Reproduced with permission from ref 74. Copyright 2018 John Wiley and Sons.

When the stoichiometric amount (SA) of ammonia was zero, spherical particles are clearly visible in Figure 6a, with an average size of 88 ± 30 nm. At 0.7 SA ammonia, the particles lose their identity, disintegrating up to some extent, and much smaller particles (<5 nm) were formed. It should be mentioned that some of these particles were aggregated as indicated by the dotted circle in Figure 6b. The average size of these aggregates was around 130–140 nm according to the cryo-TEM micrographs. This clearly indicates that upon increasing the amount of ammonia, more chains become solubilized in water, leaving the particle.

As has already been discussed above, the neutralization step is a prerequisite for the polymeric surfactants to become surface active. The static surface tension of four different neutralized polymers can be clearly seen in Figure 7. It can be seen that the surface tension is lower when the DS increases, which is directly related to the higher acid value and hence charge density of the polymers. For the same acid values, lower molar mass polymers give slightly lower surface tensions on average. According to the authors, this is related to the closer packing possibilities at the interface for these smaller molecules.

**Figure 7:**
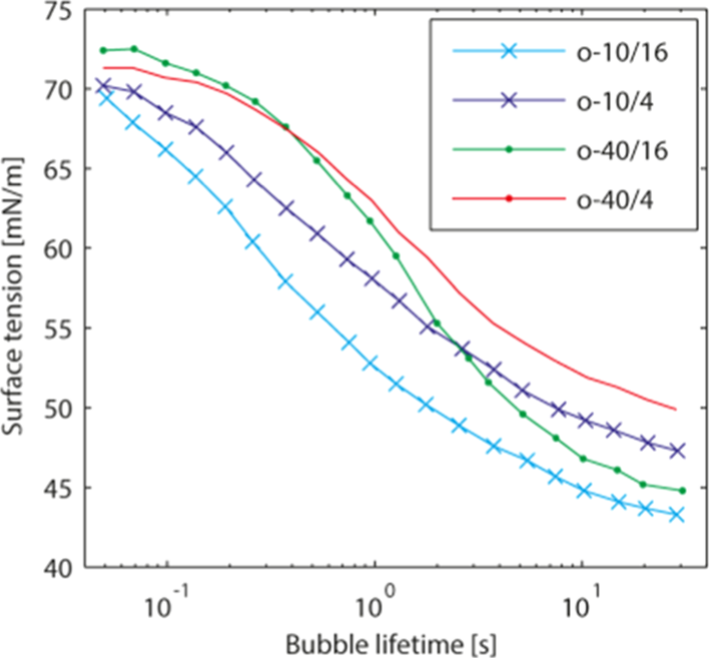
Dynamic surface tension for the polymeric surfactants neutralized with ammonia. Reproduced with permission from ref 74. Copyright 2018 John Wiley and Sons.

The polymeric surfactants with a higher molar mass are less dynamic, as is evident from their higher surface tension at low bubble lifetime.

### 3.2 Emulsification and Polymerization

One of the main advantages of synthesizing polymeric surfactants by emulsion polymerization is that they can be used as the sole stabilizers during the emulsion polymerization process step. The effectiveness of the polymeric surfactants in emulsifying monomers was demonstrated using REACT-IR.^81^ The REACT-IR probe detects small (monomer swollen) particles and water-dissolved monomers. Large monomer droplets are outside the detection range of the REACT-IR probe.

As presented in Figure 8a, the IR signal from the monomers appears around 1200 cm^–1^ and grows during a period of approximately 10 min after the addition of the monomers, indicating that monomers are emulsified by the polymeric surfactant. When the initiator is added, the polymerization starts, and the conversion of the monomers is almost instantaneous, reaching >99.9% conversion. This is also evident from the intensity vs time plot presented in Figure 8b. This particle size is between 50 and 300 nm, depending on the amount of polymeric surfactant used.

**Figure 8:**
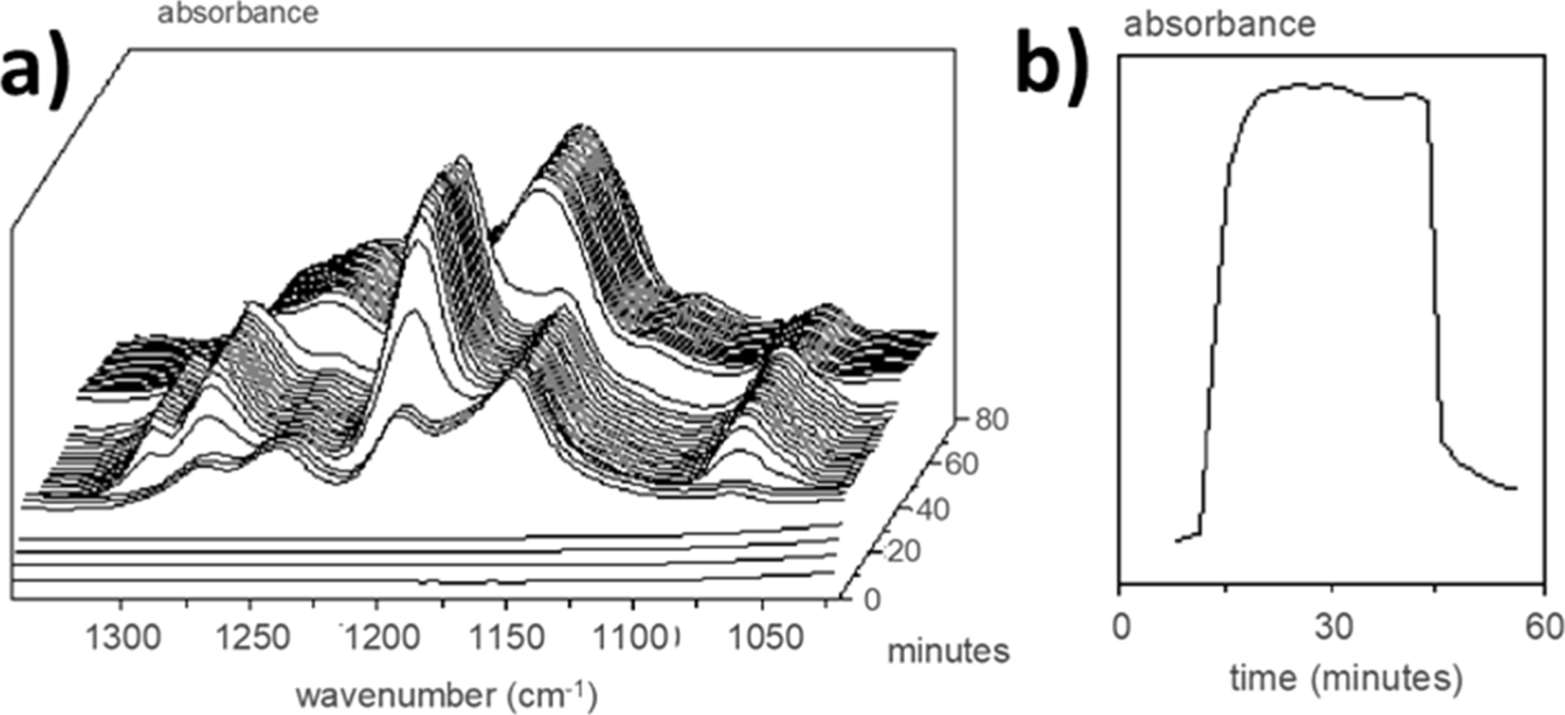
(a) React-IR traces of the emulsification of monomers by the polymeric surfactant. (b) Intensity versus time plot during emulsification. Reproduced with permission from ref 81. Copyright 2002 International Waterborne, High Solids, and Powder Coatings Symposium.

The polymerization in the presence of the polymeric surfactants can be done either by batch or semibatch process. In a batch process, which is a monomer-flooded process, a large amount of monomer is added, and the reaction mixture is stirred until all monomers are dispersed in the system, forming monomer droplets, swelling the particles formed by the polymeric surfactants, and also partially dissolving in the water phase. Then, radical initiators are added, and the polymerization starts. In a batch process, very high conversions (>99.99%) are achieved within a short period of time. However, in a semibatch process, which is typically monomer starved, both the monomers and the initiator(s) are fed over a certain time, commonly keeping high instantaneous monomer conversions and achieving really high conversions (>99.99%) as well. The choice of the process depends on several factors, and the type of polymeric surfactant is a key consideration. Typically, polymeric surfactants made via solution or bulk polymerization cannot be used in a batch process due to their limited surface activity.

It is generally assumed that when the polymeric surfactants gather to form the particles, a hairy layer is formed around the particle, which could affect the kinetics for subsequent polymerization. For instance, Kato et al. studied the effect of the poly­(MMA-comethacrylic acid) surfactant on the emulsion polymerization of styrene. Both the rate of polymerization and the number of particles increased with increasing the molar mass of the polymeric surfactant, being in the range between 5000 and 10,000 g/mol.^82^ The effect of the polymeric surfactants on the radical entry in a miniemulsion polymerization was studied by Caballero et al.^58^ and Peck and Asua.^83^ The type of monomer (MMA versus S) or initiators (APS, TBHP/AcAc, and AIBN) together with combinations of them was studied, and it was observed that all influenced the rate of polymerization. All of the mentioned studies showed that the rate of radical entry was reduced by the presence of the polymeric surfactant.

### 3.3 Film Formation Process of Binders Using Polymeric Surfactants

Regarding the morphology of the waterborne acrylic binders prepared by emulsion polymerization using polymeric surfactants, it could be said that they present a core–shell particle morphology with the polymeric surfactant on the surface stabilizing the polymer particles. Figure 9 shows a schematic representation of such a particle morphology. Typically, the shell is hydrophilic, surrounding a more hydrophobic core.

**Figure 9:**
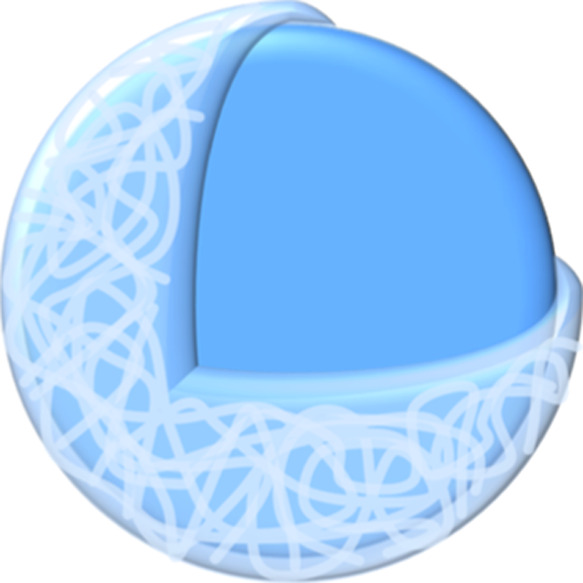
Representation of the particle morphology of a binder made using polymeric surfactants. The polymeric surfactant chains are indicated in light blue; the polymer phase made in its presence is indicated in dark blue.

The film formation of polymeric surfactant-based polymers has been studied by several authors. One important benefit of incorporating polymeric surfactants is that, depending on their acid value, they can contribute to film formation due to water plasticization.^84–87^ For example, the difference between the dry and wet *T*_g_ for a polymer composed of MMA/MAA (ionized) 90/10 (wt/wt) is 57.3 °C, as calculated according to the method described by Tsavalas and Sunberg.^84^ This makes it possible to use polymeric surfactants with high *T*_g_ without compromising film formation and particle coalescence. This was also shown by Brito and Ballard, who highlighted the importance of water plasticization of the polymeric surfactant in relation to the polymer *T*_g_ for proper film formation and coalescence.^88^

Taking advantage of the plasticizing effect of the polymeric surfactants, Qin et al.^87^ used high *T*_g_ MMA/BA/S polymeric surfactants to increase the heat resistance of the coatings. They observed that the high *T*_g_ polymeric surfactant also induces the formation of a microphase separation structure with a dual glass transition in the latex film, which improves heat resistance. In addition, the MFFT of the final polymeric dispersion was decreased, increasing the polymeric surfactant content (see Figure 10) due to the hydroplasticizing effect, which improved latex film flatness, and enhanced its glossiness.

**Figure 10:**
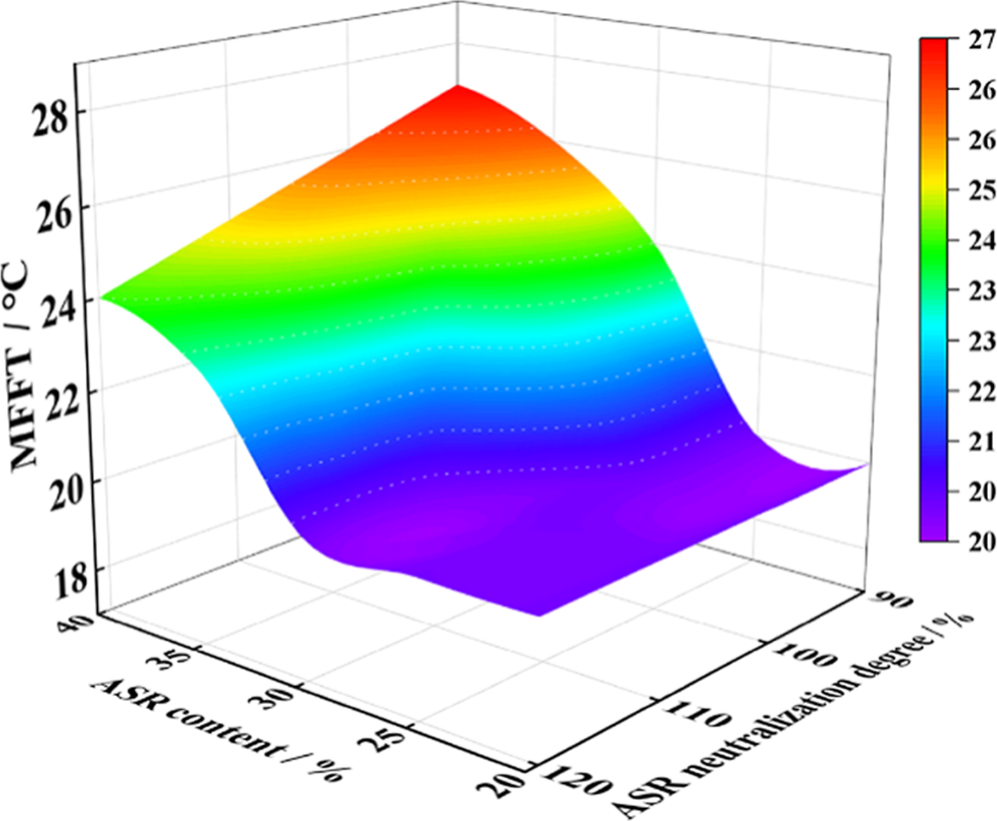
Effect on MFFT varying the polymeric surfactant content and neutralization degree. Reproduced with permission from ref 87. Copyright 2023 John Wiley and Sons.

The film formation of a blend of a polymeric surfactant and a pBMA latex was studied using atomic force microscopy (AFM).^89^ Incompatibility between the pBMA latex and the polymeric surfactant caused the polymeric surfactant to reside in the interstices between the particles and on the coating surface. It was mentioned that the polymeric surfactant hindered the coalescence of the pBMA particles.^90^ DMA analysis was used to study the effect of this incompatibility on the mechanical properties. Two different *T*_g_s were observed, and it also showed that grafting of the polymeric surfactant onto the pBMA latex improved the miscibility between the two.^91^ A more detailed study on the effect of polymeric surfactants on the film formation was undertaken by Gonzalez et al. with similar conclusions.^92^ This work used environmental scanning electron microscopy, fluorescence resonance energy transfer, and AFM to study the film formation process. They concluded that the polymeric surfactant acted as a barrier against coalescence, and this, combined with the immiscibility between the polymeric surfactant and polymer (MMA/BA), resulted in films with poor mechanical properties. However, annealing at elevated temperatures improved the mechanical properties. On the other hand, Wu et al. studied the effect of a polymeric surfactant on the paint properties of blends with soft latex particles.^93^ With this soft latex (*T*_g_ = 5 °C), film formation was not compromised up to the use of 20 wt % polymeric surfactant. The presence of the polymeric surfactant increased the tensile strength, decreased the elongation at break, deteriorated the scrub resistance, improved the block resistance, and had a negative effect on the wet adhesion.

The coating morphology derived from such dispersions is shown in Figure 11 in which the two-phase morphology is clearly visible. In this example, the soft domains represent the polymer phase that is prepared in the presence of the polymeric surfactants, which is the stiffer phase surrounding the softer domains.

**Figure 11:**
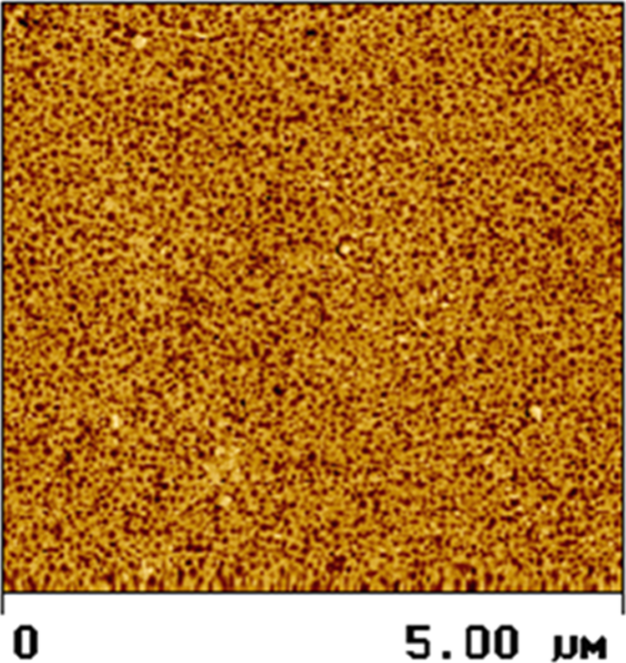
AFM of the coating morphology of a binder prepared with a polymeric surfactant. The dark areas represent the softer polymer phase; the light areas represent the stiffer, higher *T*_g_ phase. Reproduced with permission from ref 25. Copyright 2001 Vincentz Network and www.european-coatings.com.

It has already been mentioned that with the use of polymeric surfactants, leaching to the surface of the polymeric film is limited, and hence, the film performance regarding water sensitivity is improved. Bandiera et al.^73^ went a step further; they used a polymeric surfactant that could undergo hydrogen abstraction by reactive radicals from acrylic moieties, followed by propagation of the newly formed active centers and the incorporation through the polymerizable double bonds present on the backbone of the polymeric surfactant. They confirmed the grafting reaction by NMR and highlighted the possibility that this kind of polymeric surfactant may help even more, avoiding leaching during the film formation of waterborne coatings.

Very recently, Li and co-workers^94^ synthesized a cross-linked polymeric surfactant via emulsion polymerization using styrene, MMA, and MAA, along with diacetone acrylamide (DAAM). Subsequently, they synthesized polyacrylate latexes using a one-pot approach, with the aim of investigating the influence of the cross-linkable polymeric surfactant on the final film properties of the corresponding latexes. They concluded that incorporating a certain number of DAAM groups in the polymeric surfactant enhanced the adhesion of the waterborne inks in PE substrates. Zhang et al. used a similar technology but managed to produce low acid value polymeric surfactants.^95^ They also showed that combining these polymeric surfactants with traditional low molar mass surfactants had a synergetic effect. The adhesion issues related to the migration of the low molar mass surfactants were prevented, while the salt resistance increased, improving the overall performance of the inks.

## 4 Effect of Polymeric Surfactants on Performance

As discussed before, polymeric surfactants can be added to the final emulsion in quantities as high as 50%, therefore significantly contributing to the final properties of the film.

Very recently, Naderi et al. studied the use of a vinyl acetate/vinyl neodecanoate binder based on a polymeric surfactant as an additive in cement mixtures.^96^ In this application, the binders are used in dry form, but they must be redispersible in water. The binders coagulated at pH 3 and could be successfully redispersed using Ultraturrax. The acid value in the polymeric surfactant was important for ease of redispersibility. Table 1 shows a summary of different applications in which polymeric surfactants have successfully been used, indicating the chemistry of the polymeric surfactant in each case.

**Table 1:** Summary of Different Applications Where Polymeric Surfactants Have Been Used with the Type of Copolymer

application	nature of polymeric surfactant	refs
ink	styrene, α-MS, AA, styrene, MMA, (meth)acrylic acid, butyl acrylate, and/or 2-ethylhexyl acrylate	^97,98^
heat resistance	styrene–maleic anhydride copolymer	^35^
PSA	not specified	^99,100^
paper coatings	vinyl acetate, EA, methacrylic acid	^101^
leather	butyl acrylate/AA	^102^
latex gloves	hydroxyl group-containing water-soluble polymer	^103^
wall and trim paints	JONCRYL 675/JONCRYL 678 (acrylic, methacrylic acid containing resin)	^104^
barrier coating	styrene, maleic anhydride	^105^

One area where polymeric surfactant-based binders have found extensive use is in barrier coatings on paper substrates. Within the packaging industry, an important trend is the shift from plastic packaging to paper-based packaging, which is motivated by recyclability. Plastic packaging tends to consist of multiple layers of different polymers that are difficult to recycle, and a proper recycling value stream for plastic packaging is not in place. For paper, such a recycling stream is in place. However, paper as such has no barrier properties, and dispersion coatings are one approach to introduce them, for instance, barriers against water (liquid and vapor) and fatty substances.

Baker et al. studied the use of a binder based on polymeric surfactants to provide oil and water barrier properties on the paperboard. Their work showed the relevance of surface defects on both water and oil barriers, whereas the coating morphology was shown only to affect the water barrier. The importance of film formation was demonstrated by the improved performance when adding 5 wt % of isopropyl alcohol.^106,107^ Their work also showed that oil and water have different paths by which they penetrate the coatings. The main penetration of oil was via defects and pores, while for water, the chemical structure of the binder was important particularly the amount of carboxylic acid in the polymeric surfactant. The same group also studied the gas barrier on a paperboard. For a barrier against N_2_, CO_2_, and O_2_, defect-free coatings were needed, and this was obtained by applying three separate layers. For the barriers against water vapor, two layers were sufficient to achieve a moisture vapor transmission rate of 240 g/(m^2^·day). The coating could be removed by immersion into an alkaline aqueous solution, which is relevant from a recycling perspective.^108^ The use of polymeric surfactant to enable recycling of substrate was also studied by Badía et al., who applied this approach to removal of pressure-sensitive adhesives (PSA).^109^ In their study, complete removal was observed within 20 min of immersion in an alkaline solution.

In printing applications, the use of polymeric surfactants brings beneficial properties such as adhesion and heat resistance, and in particular, reversibility and wet wrinkle.^25,81^ Reversibility is the resolubilization of a dried ink in itself after the printing cylinders are turned on again. Figure 12 shows that an ink based on a binder made with a polymeric surfactant resolubilizes after 10 s, whereas an ink based on a binder made with a low molar mass surfactant does not show decent resolubilization.

**Figure 12:**
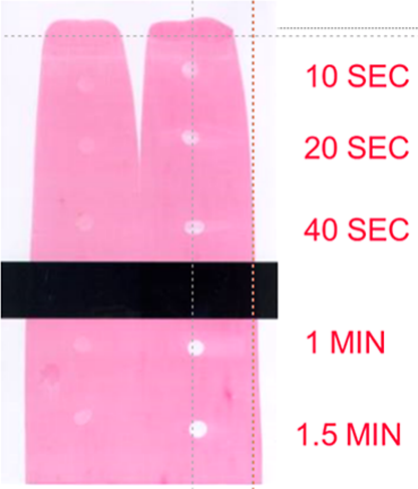
Reversibility of an ink based on a binder made with low molecular weight surfactants (left) and a binder based on a polymeric surfactant (right). The inks were dried for 1 h at room temperature before the reversibility was tested.

Another important property for packaging applications is the wet wrinkle, which is a combination of flexibility, adhesion, and water resistance. A coated substrate is divided into three parts, with one part being immersed for 1 h in water at room temperature, one part immersed in boiling water for 1 h, and the final part immersed in water and kept in the freezer for 3 h. Then, the substrates are rubbed onto themselves and the damage assessed. Figure 13 shows the performance of an ink based on a polymeric surfactant (right) versus two references that are based on low molar mass surfactants. The binder based on the polymeric surfactant outperforms the binder based on surfactants, again demonstrating the benefit of the use of polymeric surfactants.

**Figure 13:**
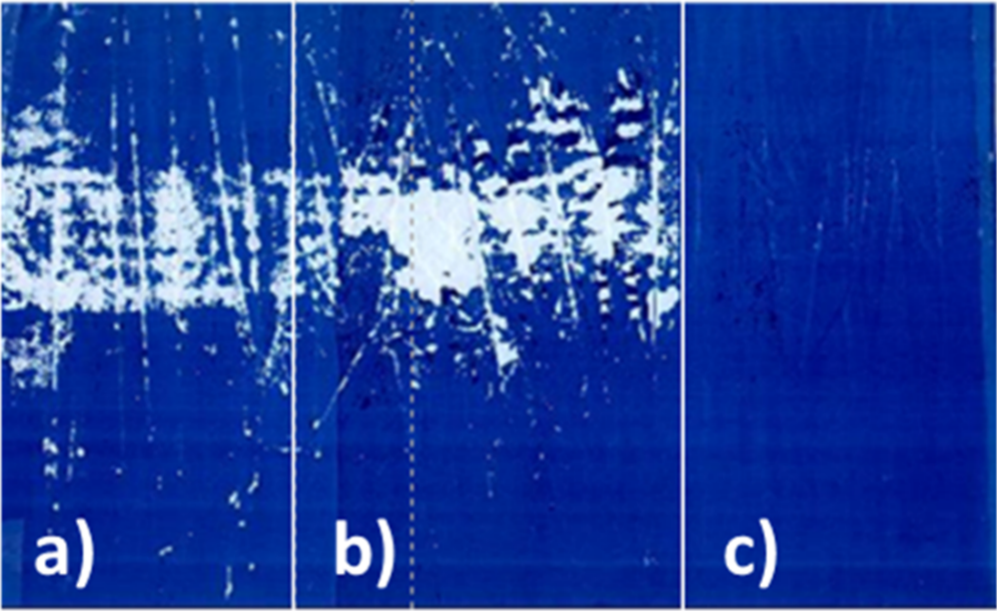
Wet wrinkle of binder based on (a) polymeric surfactants versus (b,c) surfactant-based binders.

## 5 Conclusions and Outlook

Acrylic polymeric surfactants made via emulsion polymerization are viable alternatives to traditional low molar mass surfactants. They can mitigate disadvantages of these surfactants, and, by using the emulsion polymerization steering parameters, they can be designed in such a way that they contribute to the final coating performance. This versatility has made binders based on polymeric surfactants an industrially relevant class of binders for multiple application areas, including industrial coatings, architectural coatings, and printing and packaging. Polymeric surfactants are promising candidates to play a significant role in the future, as their customizable properties could enable applications across diverse fields such as textiles, paper, electronics, personal and home care, and biomedicine. One application where this technology holds potential is recycling. Since many polymeric surfactants are soluble in alkaline conditions, an alkaline treatment can remove coatings or inks from substrates when their binders are based on these surfactants, enabling substrate recycling. This is of particular relevance for the packing industry due to the packaging and packaging waste regulation in the European Union, which requires a mandatory recycled content in packaging materials. It is expected that recycling will become important for other applications as well, such as in construction and automotive.

Last, but not least, it is clear that the polymeric surfactants offer advantages with respect to the traditional low molar mass surfactants. However, as mentioned, there are still many possibilities that these compounds offer not only the feasibility to synthesize them by emulsion polymerization but also further polymerization in the same reactor vessel. This can be beneficial for the final application of the film without compromising the industrial production of the polymeric binder.
